# The class II transactivator (CIITA) is regulated by post-translational modification cross-talk between ERK1/2 phosphorylation, mono-ubiquitination and Lys^63^ ubiquitination

**DOI:** 10.1042/BSR20150091

**Published:** 2015-07-29

**Authors:** Julie E. Morgan, Ronald L. Shanderson, Nathaniel H. Boyd, Ercan Cacan, Susanna F. Greer

**Affiliations:** * Division of Cellular Biology and Immunology, Department of Biology, Georgia State University, Atlanta, Georgia 30302, U.S.A.

**Keywords:** immunology, protein regulation, transcription regulation

## Abstract

The class II transactivator (CIITA) is known as the master regulator for the major histocompatibility class II (MHC II) molecules. CIITA is dynamically regulated through a series of intricate post-translational modifications (PTMs). CIITA's role is to initiate transcription of MHC II genes, which are responsible for presenting extracellular antigen to CD4^+^ T-cells. In the present study, we identified extracellular signal-regulated kinase (ERK)1/2 as the kinase responsible for phosphorylating the regulatory site, Ser^280^, which leads to increased levels of mono-ubiquitination and an overall increase in MHC II activity. Further, we identify that CIITA is also modified by Lys^63^-linked ubiquitination. Lys^63^ ubiquitinated CIITA is concentrated in the cytoplasm and following activation of ERK1/2, CIITA phosphorylation occurs and Lys=ubiquitinated CIITA translocates to the nucleus. CIITA ubiquitination and phosphorylation perfectly demonstrates how CIITA location and activity is regulated through PTM cross-talk. Identifying CIITA PTMs and understanding how they mediate CIITA regulation is necessary due to the critical role CIITA has in the initiation of the adaptive immune response.

## Abstract (precis)

Phosphorylation and ubiquitination cross-talk coordinate the activity of class II transactivator (CIITA), a critical adaptive immune molecule. We identify the regulatory kinase and a novel ubiquitin (Ub) modification on CIITA, Lys^63^ ubiquitination, which together act in governing the dynamic regulation of CIITA.

## INTRODUCTION

Major histocompatibility class II (MHC II) molecules are cell surface glycoproteins which present extracellular antigenic peptides to cluster of differentiation (CD)4^+^T-cells [1] in a process critical for activating adaptive immune responses [2]. MHC II expression is tightly regulated by transcriptional processes [3], initiation of which require the class II transactivator (CIITA) to be recruited to the MHC II promoter [4]. CIITA is a non-DNA-binding cofactor that binds to the components of an enhanceasome complex to initiate MHC II transcription [4]. CIITA is therefore a master regulator of MHC class II transcription and is expressed from three separate promoters, pI, pIII and pIV, each of which yields cell-specific isoforms [5]. CIITA isoform I (IF1) is primarily expressed in dendritic cells and macrophages, isoform III (IF3) is constitutively expressed in B-cells and is also interferon (IFN)-γ inducible [6,7]. IF4 is expressed from all nucleated cells and is regulated through IFN-γ induction [7].

CIITA is tightly regulated through a series of various post-translational modifications (PTMs), including acetylation, phosphorylation and ubiquitination, with IF3 in particular being heavily modified [8–14]. Previous studies in our laboratory and others reveal phosphorylation to be an essential modification to direct CIITA nuclear localization, increased transactivation and oligomerization [8,15–17]. Several residues have been identified on CIITA IF3 as sites of phosphorylation, with Ser^280^ in particular acting as a regulatory site. Once phosphorylated, mono-ubiquitination follows, leading to increased CIITA transactivity [15], enhanced association with MHC II enhanceosome components and increased MHC II transcription [15,18]. Additional phosphorylation at CIITA residues Ser^286^, Ser^288^ and Ser^293^ on IF3 by the kinase complex extracellular signal-regulated kinase (ERK)1/2 leads to the down-regulation of CIITA activity and overall nuclear export [16,19]. CIITA IF3 poly-ubiquitination has been attributed to an overall decrease in CIITA activity due to degradation via the ubiquitin-proteasome pathway [20]. Table 1 summarizes the location and consequence of known PTMs CIITA IF3 and the enzymes involved.

**Table 1:** CIITA IF3 phosphorylation and ubiquitination modifications

PTM	Modification site	Enzyme-mediating modification	Consequence(s)
Phosphorylation	Ser^280^	ERK1/2	Regulates subsequent mono-ubiquitination
Phosphorylation	Ser^286^, Ser^288^, Ser^293^	ERK1/2	Inhibits MHC II transcription and regulates nuclear export
Mono-ubiquitination	Lys^315^, Lys^330^, Lys^333^	Undetermined	Increases CIITA activation and overall MHC II expression
Lys^63^-linked ubiquitination	Undetermined	Undetermined	Involved in PTM cross-talk with ERK1/2 to mediate movement of CIITA from the cytoplasm to nucleus

Increasing evidence shows cross-talk between PTMs including phosphorylation and ubiquitination. These PTMs play roles in regulating protein location, interaction and activity [21,22]. Our previous observations of Ser^280^ phosphorylation regulating subsequent ubiquitination left the relationship between these two modifications and the enzymes involved unknown [15]. Previous observations demonstrate CIITA IF1 has a similar regulatory phosphorylation site at Ser^357^, which resides within the CIITA degron and is phosphorylated by the kinase ERK1/2 [10]. To gain better understanding of CIITA's PTM network and the intertwined roles of phosphorylation and ubiquitination in regulating CIITA activity, we investigated the effects of ERK1/2 phosphorylation at Ser^280^ and the affects it would have on CIITA's ubiquitination landscape. We identified ERK1/2 as the kinase-mediating phosphorylation of CIITA IF3 Ser^280^ leading to increased CIITA transactivation, increased levels of MHC II mRNA and ultimately increased cell surface expression of MHC II. Moreover, we have linked ERK1/2 phosphorylation to increased CIITA mono-ubiquitination and to Lys^63^ poly-ubiquitination. These findings identified the kinase responsible for initiating the cascade of the PTMs and map the cross-talk between phosphorylation and ubiquitination that regulate this dynamic master regulator. We have identified a novel ubiquitin (Ub) Lys^63^ linkage on CIITA IF3 and further show that Lys^63^ ubiquitination is an important modification for changing the subcellular localization and thus activity of the MHC II master regulator.

## MATERIALS AND METHODS

### Cell culture

COS cells (monkey fibroblast) from A.T.C.C. were maintained using high-glucose Dulbecco's modified Eagle's medium (DMEM; Mediatech) supplemented with 10% FBS, 50 units/ml of penicillin, 50 μg/ml of streptomycin and 2 mM of L-glutamine. Cells were maintained at 37**°**C with 5% CO_2._

### Plasmids

Flag–CIITA was described previously [15]. Myc-CIITA was kindly provided by Dr Jenny Ting. Myc-S280A CIITA was generated using QuickChange Lightening site-directed mutagenesis kit (Stratagene). Specific primers with desired mutations were previously described [15]. Mutagenesis reactions were performed as per the manufacturer's protocol. Mutagenesis was confirmed by sequence analysis and expression analysed by Western blot. Flag–ERK1 and Flag–ERK2 were kindly provided by Dr Michael Weber [23]. Hemagglutinin (HA)-mono-Ub and HA-poly-Ub were previously described [15]. The mono-Ub construct contains lysine mutations to arginine; all seven internal lysines of Ub are mutated (Lys^6, 11, 27, 29, 33, 48 and 63^) thus inhibiting the formation of poly-ubiquitination. HA–Lys^63^ Ub and HA–Lys^48^ Ub were gifts from Dr Ted Dawson (Addgene) [24], all other lysine residues within Ub have been mutated to arginine except Lys^48^ or Lys^63^ respectively, allowing only poly-ubiquitination to occur; i.e., where lysine residues form either Lys^48^-linked or Lys^63^-linked ubiquitination respectively. The *HLA-DRA* luciferase reporter construct was described previously [25].

### Co-immunoprecipitations

COS cells were plated at a cell density of 8×10^5^ cells/10 cm tissue culture plates. Cells were transfected with pCDNA, Myc–CIITA, Myc–S280A, Flag–ERK1 and Flag–ERK2 as indicated using GeneJuice (Merck Millipore). 18 h post transfection, cells were harvested and lysed with 1% NP40 supplemented with EDTA-free protease inhibitor (Roche) on ice. Cells were pre-cleared with mouse IgG (Sigma) and Protein G (Thermo Fisher) followed by immunoprecipitated with EZ view anti-c-Myc affinity gel beads (Sigma). Immune complexes were denatured and separated by SDS/PAGE gel electrophoresis. Gels were transferred and were individually immunoblotted with anti-Myc monoclonal antibodies (Abcam). Horseradish peroxidase (HRP) conjugates were detected with HyGlo (Denville) to determine co-immunoprecipitation (co-IP) reactions. Protein content was normalized using the Nano Photometer P-class (Implen) for equal loading on non-immunoprecipitated whole cell lysates.

### Transient transfections and phosphorylation assays

COS cells were plated at 5×10^4^ cells/well density (70% confluency) in six-well plates. Cells were transfected as indicated with either Flag–CIITA or Flag–S280A CIITA using GeneJuice according to manufacturer's instructions. Ten micromolar of U0126 (Promega) was added to indicated samples at time of transfections. Eighteen hours following transfections, cells were harvested and lysed as described above. Indicated samples were treated with λ-phosphatase (40 units/μl) lysed at 30**°**C for 1 h. Lysates were normalized for protein concentration and were separated on SDS/PAGE (5% gel) by electrophoresis. Blots were individually immunoblotted with anti-Flag monoclonal antibodies (Sigma–Aldrich). HRP conjugates were detected with Denville Hyglo.

### Transient transfections and luciferase reporter assays

COS cells were plated at 5×10^4^ cells/well density (70% confluency). Following adhesion, cells were co-transfected as indicated with *HLA-DRA*, Renilla, pcDNA, Myc–CIITA, Flag–ERK1 and Flag–ERK2 using Genejuice according to the manufacturer's instructions. Twenty-four hours following transfection, cells were lysed with 1× passive lysis buffer (Promega) supplemented with complete EDTA-free protease inhibitors. Dual luciferase assays were performed using the Lmax II^384^ (Molecular Devices) according to the manufacturer's instructions. Luciferase readings were normalized to Renilla readings for protein normalization.

### RNA expression

COS cells were plated at a cell density of 8×10^5^ cells/10 cm tissue culture plates and following adhesion, cells were transfected with pcDNA, Myc–CIITA, Myc–S280A CIITA, Flag–ERK1 and Flag–ERK2, as indicated using Genejuice. Eighteen hours following transfections, cells were harvested, washed with PBS, centrifuged at 335 ***g*** at 4°C for 5 min and nine-tenth of the cell volume was used for RNA extraction. Total RNA was prepared with 1 ml of QIAzol (Qiagen) according to the manufacturer's instructions. RNA was reconstituted in 50 μl of RNAse-free water (Sigma) and was stored at −80**°**C. An Omniscript reverse transcription kit (Qiagen) was used to reverse transcribe 1 μg of RNA into cDNA. MHC II specific anti-sense primers (Sigma) were used for reverse transcription reactions (RT) and PCR was performed using a Mastercycler thermal cycler (Eppendorf). Real-time PCR reactions were carried out on an ABI prism 7900 (Applied Biosystems) using primers and probes for MHC II [25,26] and GAPDH (glyceraldehyde-3-phosphate dehydrogenase) [27]. GAPDH RNA was used to normalize mRNA values. Presented values from real-time PCR reactions were calculated from generated standard curves from each gene in triplicate reactions and were analysed via the SDS 2.0 program. One-tenth of the cell volume was lysed in 100 μl of 1% NP40 buffer supplemented with complete EDTA-free protease inhibitors (Roche) on ice. Lysates were centrifuged and the total protein concentration was determined using the Nano Photometer P-class (Implen). Western blots confirmed equal expression of expressed plasmids.

### Flow cytometry

COS cells were plated at a cells density of 8×10^5^ cells/10 cm tissue culture plates. Following cell adhesion, cells were transfected as indicated. Seventy-two hours following transfection, cells were trypsinized and washed with cold PBS. Following the wash, nine-tenth of the cell volume was resuspended in incubation buffer [0.5% BSA in PBS (w/v)] and 10 μg of phycoerythrin (PE)-labelled anti-human *HLA-DR* (clone L243, Biolegend) antibody or PE mouse IgG2a κ isotype control antibody (Biolegend) was added and the cell suspension was rotated at 4**°**C. Following antibody incubation, cells were washed twice with PBS, fixed with 2% paraformaldehyde and were stored at 4**°**C. MHC II cell surface expression was measured by LSR Fortessa and analysed using FlowJo. All samples were analysed using 10000 events per sample. One-tenth of the cell volume harvested for flow analysis was lysed in 1% NP40 buffer containing complete EDTA-free protease inhibitors (Roche) on ice. Proteins were denatured and separated by SDS/PAGE gel electrophoresis. Gels were transferred and individually immunoblotted with anti-Myc antibody (Abcam) or anti-Flag antibody (Sigma). HRP conjugates were detected with HyGlo (Denville) to determine expression of CIITA, S280A CIITA or ERK1/2 as indicated. Proteins were normalized using the Nano Photometer P-class (Implen) to determine equal loading in lysates.

### Half-life assays

COS cells were plated at a cell density of 8×10^5^ in 10 cm tissue culture plates. Following adhesion, cells were transfected as indicated. Twenty-four hours following transfections, cells were treated with 100 μM cycloheximide for 0–8 h. Following cycloheximide treatment, cells were harvested and lysed as described above. Lysates were normalized for protein concentration. As controls, COS cells were transfected as indicated and treated with 100 μM of cycloheximide and MG132 (EMD Biosciences) for 8 h. Proteins were denatured and separated by SDS/PAGE gel electrophoresis. Blots were individually immunoblotted. Proteins were normalized using the Nano Photometer P-class (Implen) and equal loading was determined.

### Ubiquitination assays

COS cells were plated at a cell density of 8×10^5^ in 10 cm tissue culture plates. Following adhesion, cells were transfected with Myc–CIITA, Flag–ERK1, Flag–ERK2, Myc control (empty plasmid), HA–Mono Ub, HA–Poly Ub, HA–Lys^63^ Ub and HA–Lys^48^ Ub as indicated (Figure 4A). Following transfection, cells were harvested and were lysed as described above. Lysates were normalized for protein concentration. Cells were pre-cleared with Protein G (Thermo) and mouse IgG agarose beads (Sigma–Aldrich) and were immunoprecipitated with EZ view Red anti-Myc agarose beads (Sigma–Aldrich). Immune complexes were denatured and separated by SDS/PAGE gel electrophoresis. Immunoblot gels were transferred to PVDF membranes and were individually immunoblotted with anti-Ub antibody (Life Sensors). Lysate gels were transferred to nitrocellulose and were individually immunoblotted with anti-Myc (Abcam) and with anti-Flag (Sigma) antibodies. Proteins were normalized and equal loading was determined in lysates. (Figure 4C) 10 μM U0126 was added as indicated at time of transfections. Twenty-four hours following transfections, 10% FBS media was removed from indicated samples and was washed with 1× PBS and 1% FBS was added for 4 h. Hundred micromolar of MG132 was added 4 h prior to harvest, to the indicated samples. Two-hundred nanomolar PMA (Sigma) was added for 30 min to the indicated samples. Following PMA treatment, cells were harvested and lysed as above. Samples were immunoprecipitated with EZ view Red anti-Myc agarose beads (Sigma). Immune complexes were denatured and separated by SDS/PAGE gel electrophoresis. Immunoblots were transferred to PVDF membranes and were individually immunoblotted with anti-Ub antibody (Life Sensors). Lysate gels were blotted with anti-Myc (Abcam), anti-P42/44 ERK or (PhosphoSolutions), anti-actin (Cell Signaling). HRP conjugates were detected as described above. Proteins were normalized and equal loading was determined in lysates (Figure 4E). Twenty-four hours following transfections, cells were harvested and were lysed as described above. Lysates were normalized for protein concentration, pre-cleared with protein G (Thermo) and mouse IgG (Sigma) and were immunoprecipitated with EZ view Red anti-Myc agarose beads (Sigma). Immune complexes were denatured and separated as described above. Blots were immunoblotted with anti-HA (Santa Cruz Biotechnology) anti-Myc (Abcam) or anti-Flag (Sigma) antibodies. HRP conjugates were detected as described above. Proteins were normalized and equal loading was determined in the lysates.

### Cell fractionation assays

COS cells were plated at a cell density of 8×10^5^ in 10 cm tissue culture plates. Following adhesion, cells were transfected with Myc–CIITA and HA–Lys^63^ Ub as indicated. Twenty-four hours following transfection, 10% FBS media was removed, cells washed with PBS and 1% FBS DMEM media was replaced for 6 h serum starvation where indicated. Thirty minutes prior to harvest, cells were treated with 200 nM of PMA (Sigma). Following treatments, cell fractionation was completed with ProteoExtract Subcellular Proteome Extraction Kit (Calbiochem), following manufacturer's protocol. Following fractionation, proteins were normalized and immunoprecipitated with EZ view Red anti-Myc agarose beads (Sigma–Aldrich). Immune complexes were denatured and separated as described above. Blots were individually immunoblotted with anti-HA antibody (Santa Cruz Biotechnology). Lysate and fractionation controls were immunoblotted with anti-Myc (Abcam), anti-HSP 90, anti-H3 (Cell Signaling Technology), anti-p44/42 MAPK (phosphorylated mitogen-activated protein kinase; ERK1/2; Thermo Scientific) and anti-ERK1/2 rabbit polycolonal antibody (Proteintech).

### Immunofluorescence confocal imaging

COS cells were plated at a density of 5×10^4^ in six-well plates with glass coverslips. Eighteen hours following adhesion, cells were transfected with Myc–CIITA and HA–Lys^63^ Ub. Twenty-four hours following transfections, 10% FBS media was removed and washed with PBS and 1% FBS media was replaced were indicated for 6 h. Thirty minutes prior to fixation, 200 nM of PMA was added to 1% serum media to activate ERK1/2. All media was then removed and washed with PBS. To fix the cells on coverslips, ice-cold methanol was added and incubated at −20**°C** for 10 min. Methanol was aspirated and 500 μl of 2% BSA–PBS was added. To stain the coverslips, anti-CIITA (Santa Cruz Biotechnology), anti-Lys^63^ Ub (Abcam), anti-p44/42 MAPK (Thermo Scientific) or anti-ERK1/2 (Proteintech) was added to the indicated samples for 1 h. Following staining, antibody was removed and coverslips were washed 5× with PBS. Goat anti-rabbit Alexa 594 and goat anti-mouse Alexa 488 (Invitrogen) were added for 1 h in the dark. Following secondary antibody incubation, antibody was removed and samples were washed 5× with PBS. DAPI (1:1000 in PBS; Life Technologies) was added to coverslips and incubated for 10 min in the dark. DAPI was removed and coverslips were washed 5× with PBS. Coverslips were mounted with ProLong Gold Antifade (Life Technologies). Samples were allowed to dry overnight in 4**°C**. Images were obtained using the LSM 700 Confocal Microscope (Zeiss) using 40× magnification.

## RESULTS

### The kinase complex ERK1/2 associates with CIITA IF3 and phosphorylates CIITA within its degron sequence

CIITA is a large multi-dimensional protein that is tightly regulated through a series of PTMs [9,10,13–15,19,28]. Previously, we identified a regulatory phosphorylation site, Ser^280^ that lies within the degron sequence [15]. Phosphorylation at Ser^280^ leads to subsequent mono-ubiquitination on three degron proximal lysine residues, leading to increased CIITA transactivity [15]. CIITA IF1 contains a homologous serine residue (Ser^357^) that lies within a degron sequence phosphorylated by ERK1/2 [10]. We sought to determine if CIITA IF3 Ser^280^, located within the degron sequence, was similarly targeted for phosphorylation by ERK1/2. First, to determine association of CIITA and the kinase complex; a co-IP assay was performed. Expression of wild-type (WT)-CIITA with ERK1/2 in COS-7 cells, followed by co-IP, demonstrates CIITA association with the individual components of the kinase complex, ERK1, ERK2 or with the ERK1/2 complex (Figure 1A).

**Figure 1:**
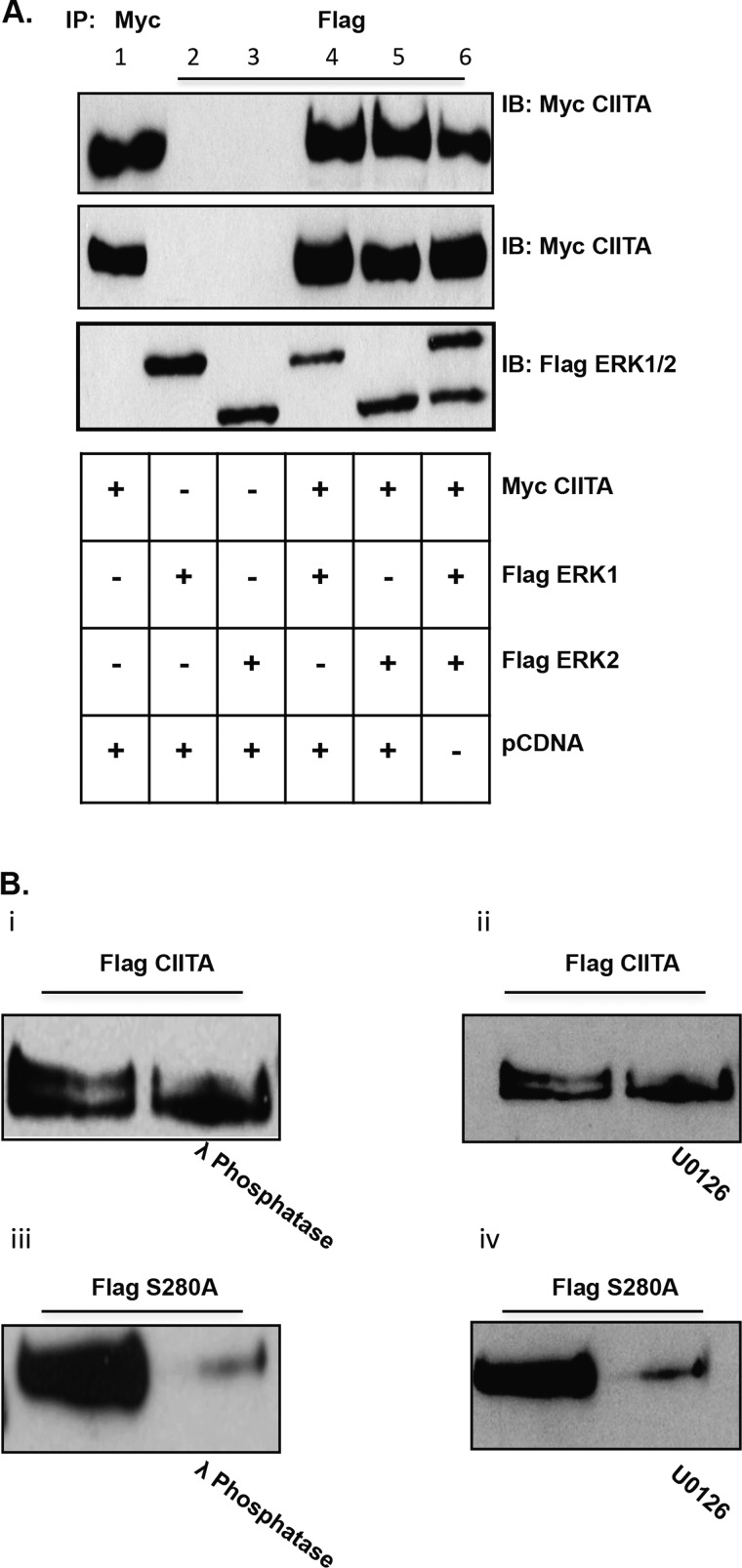
Kinase complex ERK 1/2 associates with CIITA and phosphorylates Ser^280^ (**A**) CIITA IF3 and kinase complex ERK1/2 associate. Co-IP of CIITA IF3 and ERK1/2, COS cells were co-transfected with Myc–WT–CIITA and Flag–ERK1 or Flag–ERK2 or a combination of both. Cells were harvested, lysed, pre-cleared and immunoprecipitated (IP'd) with anti-Myc or anti-Flag antibody, as indicated. Western blots were performed and IP'd samples were immunoblotted (IB) using anti-Myc antibodies. Lysate controls demonstrate expression of Myc–CIITA and Flag–ERK1/2. Data shown are cropped images from one IP gel and one lysate gel and are representative of three individual experiments. (**B**) Phosphorylated CIITA is represented by a doublet at 145 and 149 kDa (first lane in i and ii). WT–CIITA–IF3 treated with λ- phosphatase or U0126 (specific MEK inhibitor) loses the upper band (second lane in i and ii) indicating loss of phosphorylation. CIITA S280A does not migrate as a doublet (first lane in iii and iv). The CIITA S280A mutant treated with λ phosphatase or U0126 indicates loss of protein (second lane in iii and iv).

The phosphorylated form of CIITA migrates as a doublet at 145 and 149 kDa [16,19]. The upper band is indicative of the phosphorylated form (Figures 1Bi and 1Bii, first lane) [12,16,19,29]. WT-CIITA treated with λ phosphatase shows removal of phosphate groups, leaving the lower unphosphorylated form (Figure 1Bi, second lane). WT-CIITA treated with U0126, a selective MEK (MAPK/ERK kinase) inhibitor, is also void of the higher molecular mass band, indicating ERK1/2 contributes to CIITA phosphorylation (Figure 1Bii, second lane). The serine to alanine mutant of CIITA, S280A, does not show the doublet pattern (Figures 1Biii and 1Biv, first lane), when CIITA S280A is treated with either λ phosphatase or with U0126; CIITA S280A is also less stable as indicated by the decrease in protein expression (Figures 1Biii and 1Biv, second lane). The instability of the S280A CIITA upon treatment with either U0126 of λ phosphatase we believe is due, in part, to depletion of phosphate residues on other ERK1/2-mediated phosphorylation sites (Ser^286^, Ser^288^ and Ser^293^) [19]. Whereas ERK1/2 phosphorylation at these sites is known to suppress CIITA; it remains unknown if Ser^280^ phosphorylation is required in order for additional sites to be phosphorylated. When all ERK1/2 phosphorylation is blocked, it is likely S280A CIITA is a non-viable and unstable protein.

### ERK1/2 phosphorylation leads to increased CIITA IF3 activity and to increased MHC II expression

The phosphorylation regulation site Ser^280^ controls subsequent mono-ubiquitination that is required for increased CIITA transactivity and for increased MHC II transcription [15]. When CIITA Ser^280^ is mutated from serine to alanine, CIITA's activity decreases, leading to an overall decrease in the levels of MHC II expression [15]. Our observations in Figure 1 indicate CIITA is phosphorylated at residue Ser^280^ by the kinase complex, ERK1/2. To determine if the association between CIITA and ERK1/2 drives CIITA transactivation and to determine any dependence of this interaction on CIITA Ser^280^, we performed luciferase reporter assays. WT-CIITA co-transfected with ERK1/2 leads to a 2-fold increase in CIITA transactivity and ability to drive MHC II transcription; conversely, the CIITA S280A mutant has a decreased ability to drive MHC II transcription. When the CIITA S280A mutant was co-transfected with ERK1/2, CIITA transactivity levels further decrease (Figure 2A). To further identify the impact of ERK1/2, we next addressed the impact of ERK1/2 on CIITA and the Ser^280^ mutant by measuring MHC II mRNA levels in COS cells transfected with WT-CIITA or with S280A CIITA ± ERK1/2 (Figure 2B). In cells transfected with ERK1/2, MHC II mRNA levels were significantly increased, as compared with cells transfected with WT-CIITA alone. Cells transfected with CIITA S280A indicated decreased levels of MHC II mRNA as compared with cells transfected with WT-CIITA and the addition of ERK1/2 did not enhance levels of MHC II mRNA (Figure 2B).

**Figure 2:**
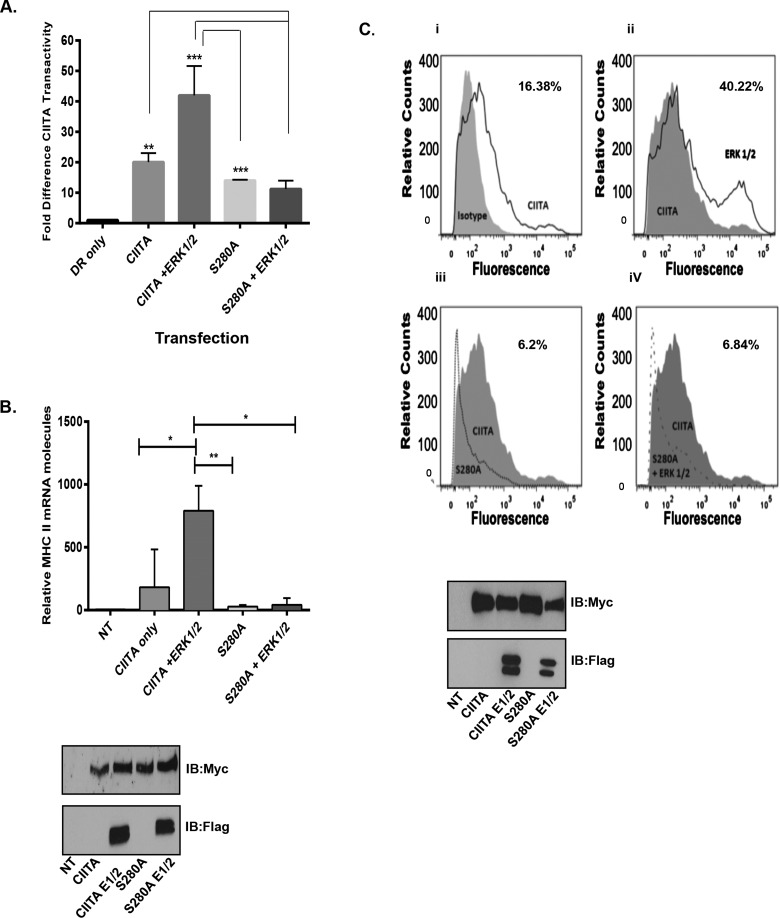
ERK 1/2 expression increases CIITA transactivity leading to increased MHC II mRNA and surface expression (**A**) CIITA transactivation increases in the presence of expressed ERK1/2. Reporter assay: COS cells were transfected as indicated with MHC II *HLA-DR-*Luc reporter construct, Renilla, CIITA, CIITA S280A and ERK 1/2. Luciferase assays were performed in triplicate in three independent experiments, data are presented as fold increase in luciferase activity. Results are standardized to Renilla values and represent the mean ± S.D. *P<0.05. (**B**) Expression of MHC II mRNA is enhanced in the presence of expressed WT CIITA and ERK 1/2. mRNA assay: COS cells were transfected as indicated with Myc–CIITA, Myc–CIITA S280A, Flag–ERK1/2. Cells were harvested, RNA extracted and cDNA was prepared and quantified by real-time PCR. Levels of MHC II mRNA were normalized to GAPDH mRNA. Data presented are results of three independent experiments and represent ± S.E.M. *P<0.05, **P<0.005. Western blots indicate equal transfection and expression of Myc–CIITA and Flag–ERK1/2. (**C**) Expression of CIITA and ERK1/2 leads to an increase in cell surface expression of MHC II. Flow cytometry: COS cells were transfected with Myc-CIITA, Myc–CIITA S280A and Flag–ERK1/2. Seventy-two hours following transfection, cells were trypsinized, washed and incubated with PE-labelled anti-human HLA-DR antibody. Following incubation, cells were fixed and PE cell surface staining was measured by LSR Fortessa. (**i**) COS cells were transfected with Myc–CIITA (dark grey line) and COS cells were stained for isotype control (grey shaded). (**ii**) COS cells were transfected with Myc–CIITA (grey shaded) and were used to compare with Myc–CIITA and Flag–ERK1/2 (grey line). (**iii**) COS cells were transfected with Myc–CIITA (grey shaded) and were compared with cells transfected with CIITA S280A (grey dotted line). (**iv**) COS cells were transfected with Myc–CIITA (grey shaded) and compared with cells transfected with Myc–CIITA S280A and Flag–ERK1/2 (grey dotted line). Western blots indicate equal transfection and expression of Myc–CIITA, Myc–CIITA S280A and Flag–ERK1/2. Results shown are representative of three separate experiments.

### Phosphorylation at Ser^280^ by ERK1/2 enhances MHC II cell surface expression

MHC II molecules are responsible for recognition of extracellular antigens [1]. Prior to MHC II surface expression CIITA is modified through various PTMs [12–16,18,19]. To further investigate the role of phosphorylation by ERK1/2 on CIITA activity, we addressed the impact of ERK1/2 on MHC II cell surface expression. Un-modified CIITA had low transactivity and lead to basal levels of MHC II surface expression of 16.38% (Figure 2Ci); WT-CIITA overexpressed with ERK1/2 enhanced MHC II surface expression to 40.22% (Figure 2Cii); transfection of the CIITA-S280A mutant in the presence and absence of ERK1/2 decreased the expression of surface expression of MHC II levels to 6.2% and 6.84% respectively (Figures 2Ciii and 2Civ).

### WT-CIITA half-life is increased when phosphorylated by ERK1/2

CIITA is constitutively expressed at basal levels in B-cells and has a half-life of 2–4 h [15]. During an active immune response, CIITA production is quickly activated to drive increased MHC II to allow B-cells to present antigen to prospective CD4^+^ T-cells [30]. Increased requirement for MHC II and therefore for CIITA requires various PTMs to CIITA [14]. ERK1/2 phosphorylates CIITA at Ser^280^; this regulatory site increases the half-life of CIITA to 6–8 h and allows a stabilized CIITA to bind the enhanceasome complex and drive the production of MHC II (Figure 3).

**Figure 3:**
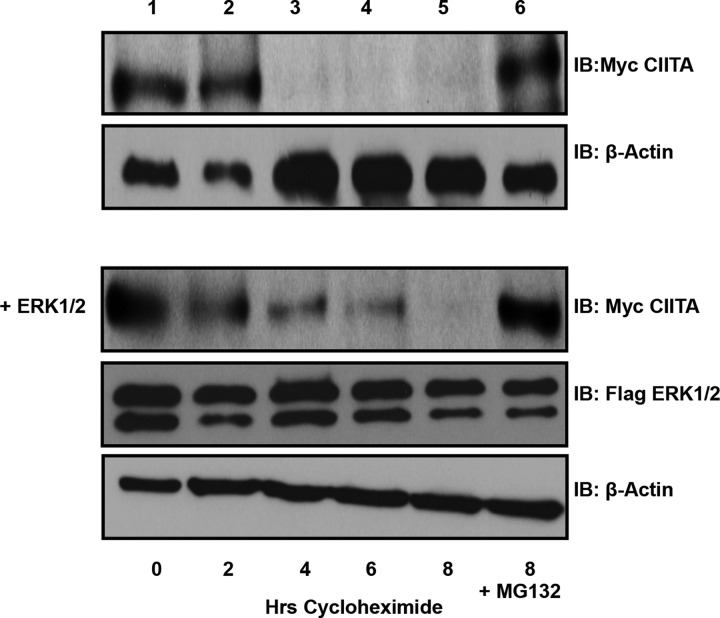
ERK1/2 expression stabilizes CIITA half-life Half-life assays: COS cells were transfected with Myc–CIITA and Flag–ERK1/2, as shown. Following transfections, cells were treated with cycloheximide for 0–8 h. Following cycloheximide treatment, cells were harvested and Western blot analysis was performed to determine the half-life of transfected Myc–CIITA (top panel). To determine effect of ERK1/2 expression on CIITA half-life, COS cells were transfected with Myc–CIITA and Flag–ERK1/2 (lower panel). As transfection and degradation controls, COS cells were transfected with Myc–CIITA and/or Flag–ERK1/2 and were treated with cycloheximide and with the proteasome inhibitor MG132 for 8 h (last lane). Experiment shown is representative of three experimental repeats.

### ERK1/2 phosphorylation of CIITA IF3 leads to an increase in global ubiquitination levels

We previously demonstrated levels of mono-ubiquitination are decreased when Ser^280^ is mutated to S280A [15]. Ser^280^ lies within the CIITA degron sequence, which is necessary for CIITA's triple mono-ubiquitination, leading to increased CIITA transactivity [15]. CIITA IF1 has a similar regulatory phosphorylation site, Ser^357^, which is phosphorylated by ERK1/2 [10]. Our data suggest Ser^280^ is also phosphorylated by ERK1/2. To determine if overexpression of ERK1/2 effects levels of global (all forms) ubiquitination, we conducted a co-IP experiment. In the absence of expressed ERK1/2, CIITA exhibits very low levels of global ubiquitination (Figure 4A, lane 1) compared with lane 2, where cells are treated with a proteasome inhibitor and CIITA exhibits maximum global ubiquitination. Alternatively, the addition of ERK1/2 results in a significant impact on CIITA global ubiquitination (Figure 4A, lane 4).

**Figure 4:**
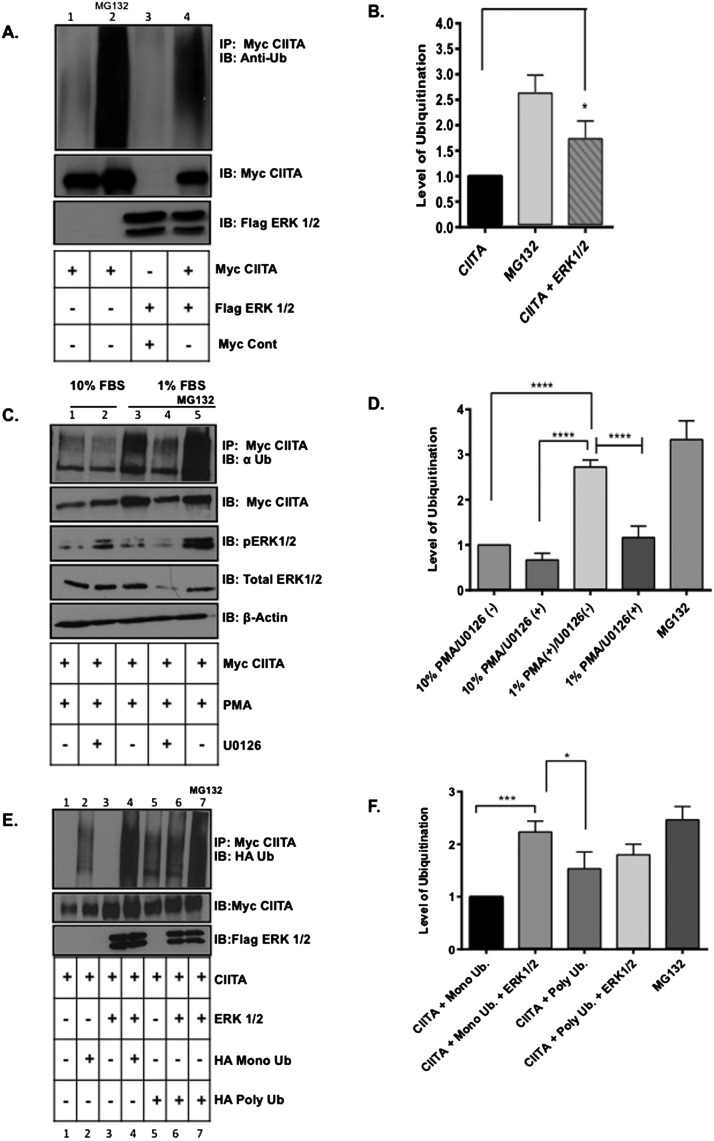
CIITA global ubiquitination and mono-ubiquitination is enhanced when ERK1/2 are overexpressed and inhibiting endogenous ERK1/2 leads to decreases in global CIITA ubiquitination levels In vivo ubiquitination assay: (**A**) COS cells were transfected with Myc–CIITA and Flag–ERK1/2. Lysate controls (bottom two panels) demonstrate expression of Myc–CIITA and Flag–ERK1/2. Data shown are cropped images from one IP gel and one lysate gel and are representative of three experiments. (**B**) Densitometry and quantification of data in Figure 4(A): Densitometry was performed on three independent experiments, ± S.D., *P<0.05. (**C**) COS cells were transfected with Myc–CIITA and indicated samples were treated with U0126 (MEK specific inhibitor) at time of transfections. Eighteen hours following transfections, 10% FBS media was replaced with 1% FBS media for 6 h and PMA and MG132 were added as indicated. Co-IP and ubiquitination analysis: Following all treatments, cells were harvested, lysed, pre-cleared and IP'd (immunoprecipitated) with anti-Myc antibodies. Lysate controls (bottom four panels) demonstrate expression of Myc–CIITA, total ERK1/2, phosphorylated ERK1/2 and actin as controls. Data shown are cropped images from one IP gel and one lysate gel. (**D**) Densitometry and quantification of data in Figure 4(C): Densitometry was performed on three independent experiments, ± S.D., ****P<0.0001. (**E**) COS cells were transfected with Myc–CIITA, Flag–ERK1/2, HA–mono-Ub or HA–poly-ubiquitin. MG132 was added to control sample for 4 h. Lysate controls (bottom two panels) demonstrate expression of Myc–CIITA and Flag–ERK1/2. Data shown are cropped images from one IP gel and one lysate gel. (**F**) Densitometry and quantification of data in Figure 4(E): Densitometry was performed on three independent experiments, ± S.D., ***P<0.001.

### Inhibiting endogenous ERK1/2 leads to a decrease in CIITA IF3 global ubiquitination levels

To investigate levels of CIITA ubiquitination when endogenous ERK1/2 is inhibited, COS-7 cells were transfected with Myc–CIITA. In cells incubated with 10% serum media and treated with PMA 18 h post-transfection, CIITA ubiquitination is very low (Figure 4C, lane 1) compared with cells serum starved with 1% serum media for 6 h followed by 30 min of PMA stimulation to activate ERK1/2 (Figure 4C, lane 3). When treated with MEK inhibitor U0126, ubiquitination levels decrease (Figure 4C lanes 2 and 4).

### ERK1/2 leads to increased levels of CIITA IF3 mono-ubiquitination

The Ser^280^ regulatory phosphorylation site on CIITA is necessary for subsequent triple mono-ubiquitination events leading to increased CIITA transactivation [15]. To determine if expression of ERK1/2 alters the levels of CIITA mono- or poly-ubiquitination, *in vivo* ubiquitination assays were performed. Expression of CIITA with HA-mono-Ub and Flag–ERK1/2 significantly increase CIITA mono-ubiquitination levels (Figure 4E, compare lanes 2 and 4; quantified in Figure 4F). To determine if ERK1/2 alters CIITA poly-ubiquitination, cells were co-transfected as indicated with Myc–CIITA, HA–poly-Ub and Flag–ERK1/2; only a small increase in CIITA poly-ubiquitination was observed over cells in which Flag–ERK1/2 was not expressed (Figure 4E, compare lanes 5 and 6; quantified in Figure 4F). Western blots demonstrate stable expression of Myc–CIITA even upon polyubiquitination, indicating the poly-Ub linkage is not exclusively Lys^48^-linked ubiquitin.

### Lys^63^ CIITA IF3 ubiquitination is increased when ERK1/2 is overexpressed

Protein degradation is often signaled via the addition of Lys^48^-linked ubiquitination on to the targeted protein [31]. Our data suggest (Figure 4E) that CIITA polyubiquitination is increased in the presence of expressed ERK1/2, but no change in CIITA expression indicates the Ub linkage is likely not driving CIITA degradation. Lys^63^ ubiquitination has recently been implicated in transcription factor regulation [32–35]. As we and others have previously shown CIITA to be phosphorylated and ubiquitinated, we next performed ubiquitination assays to determine if the ubiquitination present on CIITA in the presence of expressed ERK1/2 is an alternative linkage to Lys^48^ [15]. In the presence of expressed ERK1/2, levels of CIITA Lys^63^ ubiquitination increased significantly as compared with cells transfected with CIITA and HA–Lys^63^ Ub alone (Figure 5A, compare lanes 1 and 2 and quantification in Figure 5B). As ERK1/2 also phosphorylates CIITA at additional downstream sites that ultimately lead to the down-regulation in CIITA activity and to CIITA degradation [16,19], we also investigated if ERK1/2 would affect levels of Lys^48^-linked ubiquitination. CIITA Lys^48^-linked ubiquitination was not significantly increased in the presence of ERK1/2 over CIITA cells expressing CIITA HA–Lys^48^ alone (Figure 5A, compare lanes 3 and 4 and quantified in Figure 5B).

**Figure 5:**
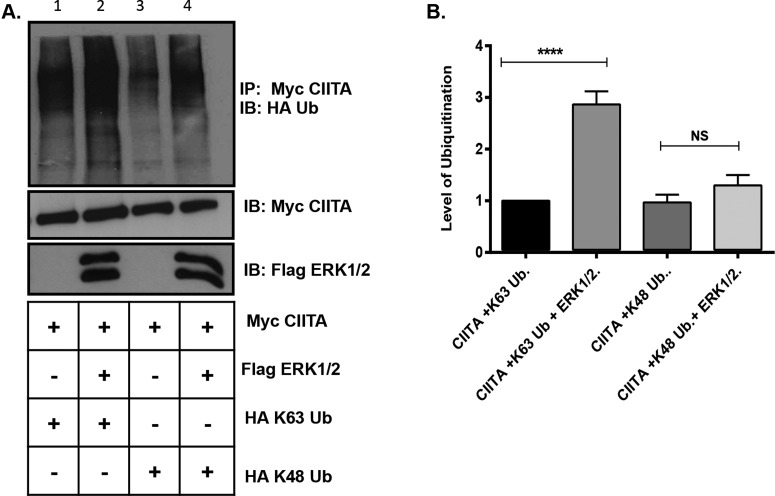
Lys^63^-linked ubiquitination increases on CIITA in presence of overexpressed ERK1/2 (**A**) COS cells were transfected with Myc–CIITA, Flag–ERK1/2, HA–Lys^63^ Ub and HA–Lys^48^ ubiquitin. Lysate controls (bottom two panels) demonstrate expression of Myc–CIITA and Flag–ERK1/2. Data shown are cropped images from one IP gel and one lysate gel. (**B**) Densitometry and quantification of data in Figure 5(A): Densitometry was performed on three independent experiments, ± S.D. ****P<0.0001.

### Lys^63^ ubiquitinated CIITA is cytoplasmic; ERK1/2 activation drives CIITA nuclear mobilization

Data from Figure 5(A), show an overall increase in Lys^63^ ubiquitination in presence of overexpressed ERK1/2. To better understand the role for Lys^63^ CIITA ubiquitination in the presence of activated ERK1/2, we conducted a cellular fractionation assay followed by co-IP. We co-transfected COS-7 cells with Myc–CIITA and HA–Lys^63^, followed by serum starvation and PMA treatment to activate the ERK1/2 kinase complex. Prior to ERK1/2 activation (without serum starvation and PMA), CIITA was highly modified by Lys^63^ Ub in the cytoplasmic fraction (Figure 6A, cytoplasmic fraction, lane 1) whereas little Lys^63^ modified CIITA is observed in the nuclear fraction (Figure 6A, nuclear fraction, lane1). When cells were serum starved and treated with 200 nM of PMA, CIITA Lys^63^ Ub levels were significantly decreased in the cytoplasmic fraction (Figure 6A, cytoplasmic fraction, compare lanes 1 and 2), but significantly increased in the nuclear fraction (Figure 6A, nuclear fraction, compare lanes 1 and 2) as shown in the densitometry analysis (Figure 6C). Equal loading, transfection and cellular fractionation are shown by the lysate controls (Figure 6B). To further investigate the changes in Lys^63^ ubiquitination observed in the cellular fractionation assay, we performed confocal microscopy. Cells were treated with 10% serum media where ERK1/2 is not activated (Figures 7G–7I). As with the fractionation assay, when 10% serum media is removed and replaced with 1% serum media and treated with PMA, ERK1/2 is phosphorylated and therefore, activated (Figures 7J–7L). CIITA was primarily located in the cytoplasm prior to ERK1/2 activation (Figures 7A–7C). Following serum starvation for 6 h and treatment with PMA for 30 min, ERK1/2 was activated and thus able to phosphorylate CIITA. Following this event, CIITA was primarily located in the nucleus (Figures 7D–7F). Previous data (Figures 5A and 6A) demonstrate CIITA is modified via Lys^63^ ubiquitination. In the presence of ERK1/2 activation (serum starvation and PMA treatment), CIITA and Lys^63^ Ub co-localize to the nucleus (Figures 7Q–7T) as compared with inactivated ERK1/2 (10% serum and no PMA) and CIITA and Lys^63^ Ub both appear in the cytoplasm (Figures 7M–7P). The merge of nuclear staining shows CIITA and Lys^63^ Ub co-localize, mirroring the results seen in the fractionation assay.

**Figure 6:**
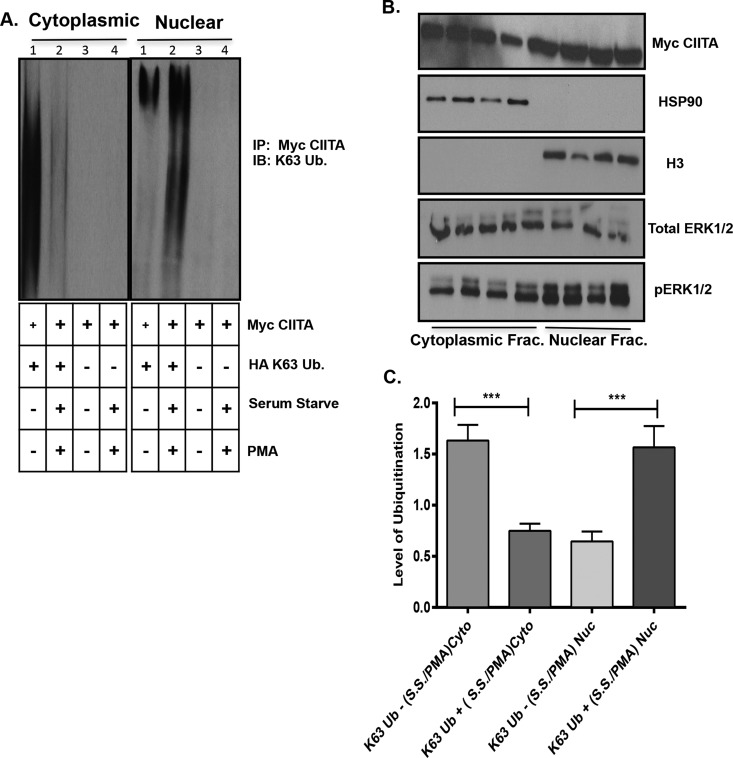
Activation of ERK1/2 leads to movement of Lys^63^-linked ubiquitinated CIITA from the cytoplasm to the nucleus Cell fractionation and IP: (**A**) COS cells were transfected with WT Myc–CIITA and HA–Lys^63^ Ub where indicated. Eighteen hours following transfections, media containing 10% serum was removed where indicated for 6 h. Thirty minutes prior to harvest, 200 nM of PMA was added as indicated. Media was removed from cells, washed and cells were fractionated and samples were normalized for protein concentration. All samples were then immunoprecipitated (IP'd) with anti-Myc antibody. Data shown are cropped images from one IP gel. (**B**) Western blots demonstrate equal expression of Myc–CIITA, total ERK1/2, phosphorylated ERK1/2, HSP90 (heat-shock protein 90; cytoplasmic fraction) and H3 (nuclear fraction). (**C**) Densitometry and quantification of data in Figure 6(A): Densitometry was performed on three independent experiments, ± S.D., ***P<0.001.

**Figure 7:**
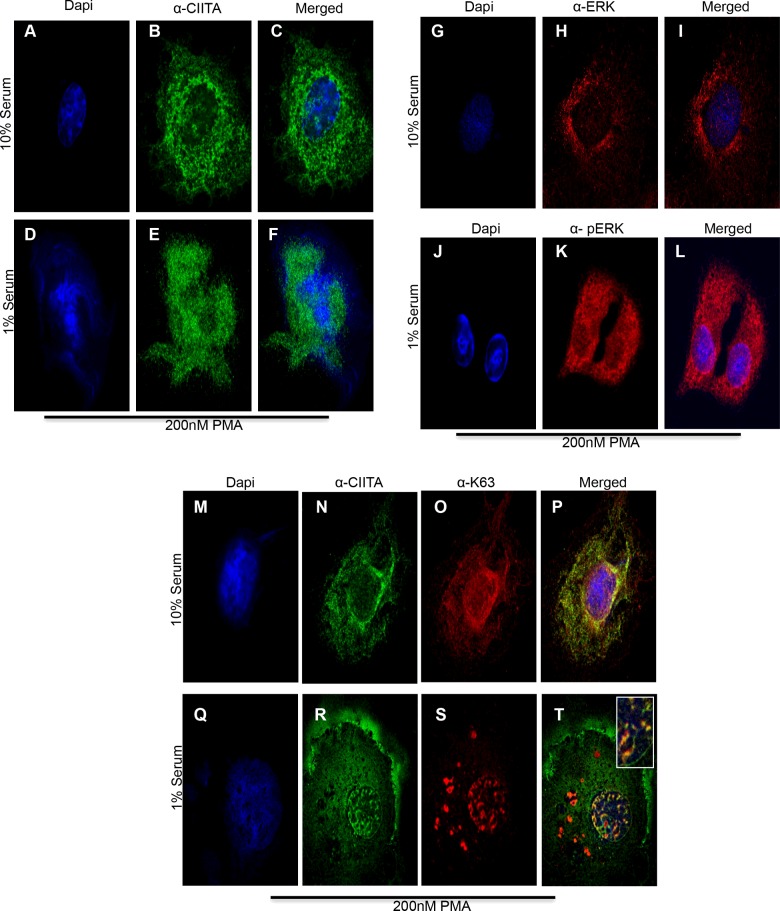
Lys^63^ Ub co-localizes with CIITA in cytoplasm and upon ERK1/2 activation moves to nucleus Immunofluorescence: COS cells were plated in six-well plates with glass coverslips. Twenty-four hours post seeding, cells were transfected with Myc–CIITA and HA–Lys^63^ ubiquitin. Eighteen hours following transfections, 10% serum media was removed where indicated and was replaced with 1% serum media for 6 h. Thirty minutes prior to harvest, 200 nM PMA was added to the indicated samples. Cells were fixed in ice-cold methanol for 10 min and were stained with anti-CIITA, anti-Lys^63^, anti-ERK1/2 and anti-pERK1/2 as indicated. (**A**–**C**) Cells in 10% serum media, stained for DAPI, CIITA and merge. (**D**–**F**) Cells in 1% serum free media, stained for DAPI, CIITA and merge. (**G**–**I**) Serum media (10%), stained for DAPI, ERK1/2 and merge. (**J**–**L**) Serum free media (1%), stained for DAPI, pERK1/2 and merge. (**M**–**P**) Serum media (10%), stained for DAPI, CIITA and Lys^63^ Ub and merge. (**Q**–**T**) Serum free media (1%), stained for DAPI, CIITA and Lys^63^ Ub and merge.

## DISCUSSION

We sought in the present study to identify the kinase responsible for phosphorylating CIITA IF3, the isoform seen constitutively expressed in B-cells, at the regulatory site Ser^280^ and to further determine the ubiquitination conjugation driven by CIITA phosphorylation. Phosphorylation and ubiquitination are both dynamic and reversible PTMs that lead to alterations of target proteins. Phosphorylation allows for changes in protein–protein interactions, localization and activity. Ubiquitination has traditionally been known for its role in tagging proteins for eventual degradation via the 26S proteasome [31]. Ubiquitination has been shown to have non-proteolytic roles including receptor internalization, multi-protein complex assembly and intracellular trafficking [36]. Phosphorylation and ubiquitination have been shown to regulate post-translational cross-talk; NF-κB (nuclear factor kappa-light-chain-enhancer of activated B-cells), cyclin E and EGFR (epidermal growth factor receptor) have all been shown to be regulated through multiplexed cross-talk [22]. In the present study, we have identified the kinase responsible for phosphorylating CIITA IF3 Ser^280^ [15]. Phosphorylation at Ser^280^ leads to non-degradative ubiquitination and increases CIITA's IF3 transactivity [15].

We and others have shown the interaction between CIITA IF3 and the kinase complex ERK1/2 [19]. Phosphorylated CIITA appears as a doublet, with the upper band representing phosphorylated CIITA [16,19]. The mutation of serine to alanine on residue 280 (S280A) shows a lack of phosphorylation at that particular site and further phosphorylation does not occur on CIITA (Figures Biii and Biv). Ser^280^ is therefore critical for additional phosphorylation to occur on CIITA. Treatment with U0126 prohibits ERK1/2 phosphorylation and the S280A CIITA IF3 mutant is unstable (Figures 1Bii and 1Biv). Previous findings identify ERK1/2 as kinases responsible for phosphorylation of CIITA at residues Ser^286^, Ser^288^ and Ser^293^, leading to down regulation of CIITA activity and eventual export from the nucleus [19]. Voong et al. [19] showed that inhibition of ERK1/2 leads to an increase in CIITA's transactivity; however, we failed to obtain similar results. These differences may be attributed to the activities of additional kinases, such as cell division cycle protein 2 (Cdc2), which was also shown to phosphorylate CIITA. Additionally, ERK5 kinase phosphorylates similar targets as ERK1/2 when ERK1/2 is unavailable [37]. Finally, Voong et al. [19] neither confirm ERK1/2 expression nor activation in assays used to determine CIITA transactivity; therefore, it is difficult to directly compare our results. We now demonstrate phosphorylation by ERK1/2 on the regulatory site Ser^280^; it is not only necessary, but also critical for CIITA transactivity. Phosphorylation of Ser^280^ by ERK1/2 leads to significant increase in MHC II mRNA and cell surface expression (Figures 2B and 2C) leading to an increased ability to present antigens.

The regulation of CIITA is accomplished through a series of dynamic PTM's [14–16,18]. The phosphorylation of CIITA by ERK1/2 leads to a sustained half-life and thus an active CIITA for a greater period of time. Active CIITA leads to increased MHC II and an immune response. Ours and other reports identify ERK1/2 phosphorylation on various sites throughout CIITA and indicate multiple roles for ERK1/2 phosphorylation in regulating activation and suppression of CIITA [19]. Although there is still a great deal to uncover in regards to phosphorylation of CIITA, including the order in which modifications are made, data clearly indicate ERK1/2 targets multiple sites on CIITA and that this kinase complex is necessary in regulating overall activity of CIITA.

Previously we linked CIITA phosphorylation at Ser^280^ and CIITA mono-ubiquitination in regulation of CIITA transactivity. To further our insight into the mechanisms regulating phosphorylation/ubiquitination cross-talk on CIITA, we investigated the influence of ERK1/2 on CIITA global ubiquitination level. Levels of global ubiquitination increase when ERK1/2 is overexpressed (Figure 4A). Furthermore, inhibition of endogenous ERK1/2 with U0126, a specific inhibitor for MEK, results in reduction in global ubiquitination. These findings indicate links between ubiquitination and phosphorylation as previously observed by our group [15]. Several reports link phosphorylation and ubiquitination in regulating transcription factors, such as FOXO4 (forkhead box O), NF-κB and cyclin E [22].

Ubiquitination is a diverse modification that yields multiple types of poly-Ub conjugates and mono-ubiquitination. Poly-ubiquitination is determined by the Ub lysine residue added to a target protein (Lys^6^, Lys^11^, Lys^27^, Lys^29^, Lys^33^, Lys^48^ and Lys^63^) in which an additional Ub molecule is attached to the already existing Ub on the target protein [31]. Our data demonstrate CIITA is modified with a different Ub linkage (Figure 4C, first blot) than either mono-ubiquitination or Lys^48^-linked ubiquitination based on the lack of protein degradation of CIITA (Figure 4A, second blot). Additionally, we show an increase in CIITA protein concentration when ERK1/2 is activated following serum starvation and treatment with PMA (Figure 4C, second blot). Together, these data suggest CIITA phosphorylation by ERK1/2 participates in PTM cross-talk with a unique ubiquitination modification, leading to increased CIITA transactivity.

The process of ubiquitination has been heavily studied; it is well known that the type of Ub linkage added to a target protein determines a proteins fate. Lys^48^-linked ubiquitination tags proteins for degradation via the 26S proteasome [31]. Lys^63^ ubiquitination of TNFR (tumour necrosis factor receptor)-associated factor 6 (TRAF6) links interleukin-1 receptor (IL-1R) signalling to the activation of NF-κB [38]. TRAF6 modification by Lys^63^ ubiquitination allows additional proteins, including TAB2 (TGF-beta activated kinase 1/MAP3K7 binding protein 2), to bind directly to the Lys^63^ Ub chain resulting in additional downstream effects including phosphorylation and nuclear translocation [32,33,39–41]. IL-1R-associated kinase 1 (IRAK1) is also modified with Lys^63^ ubiquitination, resulting in multiple protein–protein interactions and downstream phosphorylation events in the NF-κB pathway [33]. In both cases, Lys^63^ ubiquitination brings two-protein complexes together to act as a scaffold. Lys^63^ ubiquitination has also been implicated in endocytosis, protein trafficking and DNA damage repair [42]. Our observation of Lys^63^ ubiquitination is the first of any isoform of CIITA and, in the present study, we show CIITA Lys^63^ ubiquitination followed by phosphorylation by ERK1/2, which leads to cellular localization changes (Figure 5A). Additionally, we also note increased levels of CIITA Lys^48^ ubiquitination (Figure 4E), observations in line with previous reports in which ERK1/2 have been indicated in phosphorylating Ser^286^, Ser^288^ and Ser^293^ leading to the down-regulation of CIITA [16].

To gain insight into the role of Lys^63^ ubiquitination in regulating CIITA activity, we conducted cellular fractionation assays. Our data show heavily modified Lys^63^ ubiquitinated CIITA in the cytoplasm followed by a decrease once CIITA has been phosphorylated by ERK1/2 (Figure 6A, cytoplasmic fraction). The nuclear fraction shows the inverse pattern with an increase in the amount of Lys^63^ ubiquitinated CIITA, following phosphorylation by ERK1/2 (Figure 6A, nuclear fraction). Similar trends were seen in immunofluorescence images (Figures 7P and 7T). Lys^63^ ubiquitinated CIITA was predominately located in the cytoplasm and, following ERK1/2 activation, Lys^63^ ubquitinated CIITA translocate to the nucleus. When the images are merged, yellow punctate loci can be seen. In sum, these data support Lys^63^ ubiquitination as an important modification for phosphorylation of CIITA by ERK1/2. Several scenarios exist; Lys^63^ ubiquitination could act as a scaffold driving CIITA nuclear localization where ERK1/2 then binds and phosphorylates CIITA or Lys^63^ ubiquitination could be assisting in the activation of ERK1/2, as has been seen with other kinases, as is the case with protein kinase B (Akt) [42].

Because CIITA is dynamically regulated by multiple PTM's and the intertwined nature of these modifications has yet to be fully defined, it is important to decipher these modifications to gain a better understanding of the regulation of this critical adaptive immune protein. Phosphorylation of CIITA occurs on various residues [12,16,28,29]. Numerous kinases are involved in the regulation of these phosphorylation events; however, our data also indicate CIITA is regulated by the same kinase at multiple locations, but with very different impacts on protein activity. Although links between phosphorylation and mono-ubiquitination have previously been identified, the kinase responsible for initiating the chain of reactions was unknown [15]. We identify here CIITA is phosphorylated by ERK1/2 at residue Ser^280^, leading to a significant increase in CIITA mono-ubiquitination resulting in increased CIITA activity and MHC II expression and activity. Additionally, we are the first one to show a novel ubiquitination linkage on CIITA, Lys^63^. Further, we demonstrate a novel cross-talk between phosphorylation by ERK1/2 and Lys^63^ ubiquitination of CIITA IF3 and the combined role of these PTMs have in facilitating movement of CIITA from the cytoplasm to the nucleus and overall increase in activity (Figure 8). Our findings contribute to a greater understanding of the regulation of IF3 that is derived from CIITA pIII; however, as we utilized an overexpression system, the data contained within will need to be recapitulated in B-cells in order to further validate the findings observed. Relevant clinical applications of our findings include, activating CIITA PTMs are largely mediated by ERK1/2 phosphorylation. Based on these observations, dysregulation in the MAPK pathway can contribute to the development of many autoimmune diseases [43]. Although the immune effects are poorly understood, MEK inhibitors provide promising novel therapeutic approaches for the treatment of many autoimmune diseases [44]. Future work will focus on identifying the E3 ligases involved in ubiquitination of CIITA. Although there have been strides in mapping CIITA's PTMs network, considerable ground remains to be covered in understanding the web of modifications and the cross-talk occurring to regulate this ‘master regulator’ of MHC II genes and the adaptive immune response.

**Figure 8:**
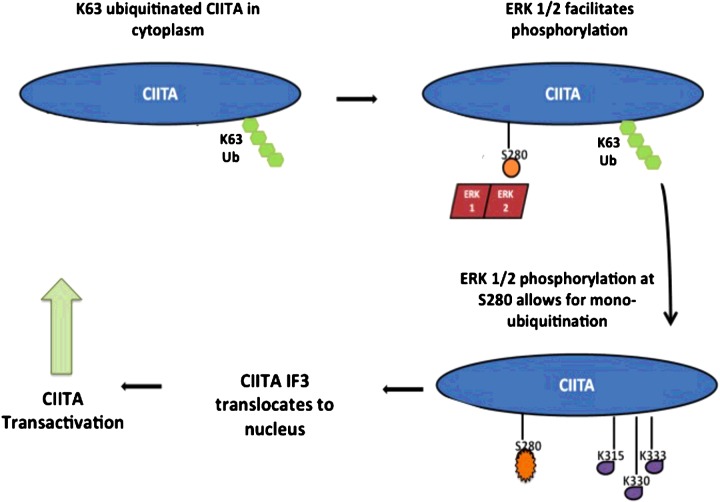
We propose CIITA IF3 is modified by Lys^63^-linked ubiquitination while in the cytoplasm by an unknown E3 Ub ligase Lys^63^-linked ubiquitinated CIITA is phosphorylated by the kinase complex, ERK1/2 at Ser^280^, which allows for CIITA to translocate to the nucleus where CIITA acts as the master regulator for the MHC II genes. It is clear that mono-ubiquitination is necessary for CIITA transactivity; however, it is unclear if mono-ubiquitination occurs in the cytoplasm or nucleus.

## Abbreviations

CD: cluster of differentiation
CIITA: class II transactivator
co-IP: co-immunoprecipitation
DMEM: Dulbecco's modified Eagle's medium
ERK1/2: extracellular signal-regulated kinase 1/2
HA: hemagglutinin
IF1/3: isoform I/III
IFN: interferon
IL-1R: interleukin-1 receptor
GAPDH: glyceraldehyde-3-phosphate dehydrogenase
HRP: horseradish peroxidase
MAPK: mitogen-activated protein kinase
MEK: MAPK/ERK kinase
MHC II: major histocompatibility class II
NF-κB: nuclear factor kappa-light-chain-enhancer of activated B-cells
PE: phycoerythrin
PTM: post-translational modification
TRAF6: tumour necrosis factor receptor-associated factor 6
Ub: ubiquitin
WT: wild-type
